# Molecular Relatedness of Maternal and Neonatal Multidrug-Resistant Gram-Negative Colonization Isolates in Low- and Middle-Income Countries: A Systematic Review

**DOI:** 10.1093/ofid/ofag010

**Published:** 2026-01-14

**Authors:** Joycelyn A Dame, Gabrielle Obeng-Koranteng, Nathan L’Etoile, Evelyn Amoah, Susan E Coffin, Adam J Ratner, Jonathan Strysko, Jiayin Zheng, Andrew P Steenhoff, Grace St. Cyr

**Affiliations:** Department of Child Health, University of Ghana Medical School, Accra, Ghana; Department of Child Health, Korle Bu Teaching Hospital, Accra, Ghana; Department of Child Health, Korle Bu Teaching Hospital, Accra, Ghana; Division of Infectious Diseases, Department of Pediatrics, Children's Hospital of Philadelphia, Philadelphia, Pennsylvania, USA; Center for Microbial Medicine, Children's Hospital of Philadelphia, Philadelphia, Pennsylvania, USA; Department of Child Health, University of Ghana Medical School, Accra, Ghana; Department of Child Health, Korle Bu Teaching Hospital, Accra, Ghana; Division of Infectious Diseases, Department of Pediatrics, Children's Hospital of Philadelphia, Philadelphia, Pennsylvania, USA; Department of Pediatrics, Perelman School of Medicine, University of Pennsylvania, Philadelphia, Pennsylvania, USA; Division of Infectious Diseases, Department of Pediatrics, NewYork University, New York, New York, USA; Department of Pediatrics, Perelman School of Medicine, University of Pennsylvania, Philadelphia, Pennsylvania, USA; Division of Pediatric Hospital Medicine, Department of Pediatrics, Children's Hospital of Philadelphia, Philadelphia, Pennsylvania, USA; Department of Pediatric and Adolescent Health, Faculty of Medicine, University of Botswana, Gaborone, Botswana; Department of Biostatistics, Epidemiology and Informatics, Perelman School of Medicine, University of Pennsylvania, Philadelphia, Pennsylvania, USA; Department of Biostatistics, Epidemiology and Informatics, Children's Hospital of Philadelphia, Philadelphia, Pennsylvania, USA; Division of Infectious Diseases, Department of Pediatrics, Children's Hospital of Philadelphia, Philadelphia, Pennsylvania, USA; Department of Pediatrics, Perelman School of Medicine, University of Pennsylvania, Philadelphia, Pennsylvania, USA; Division of Infectious Diseases, Department of Pediatrics, Children's Hospital of Philadelphia, Philadelphia, Pennsylvania, USA; Center for Microbial Medicine, Children's Hospital of Philadelphia, Philadelphia, Pennsylvania, USA

**Keywords:** low- and middle-income countries, multidrug-resistance, neonate, vertical transmission

## Abstract

**Background:**

Multidrug-resistant gram-negative (MDR-GN) sepsis is a significant cause of neonatal mortality in low- and middle-income countries (LMICs). The role of vertical transmission in neonatal MDR-GN colonization and thereby invasive infection remains unknown. The aim of this study was to systematically review literature detailing the molecular relatedness of maternal and neonatal MDR-GN colonization isolates in LMICs, characterizing the extent of vertical transmission.

**Methods:**

Following PRISMA guidelines, PubMed and Scopus databases were searched for LMIC literature reporting molecular evidence of MDR-GN concordance for mother-neonate dyads.

**Results:**

Of 90 articles identified by the search, 11 met inclusion criteria. Findings demonstrated substantial MDR-GN colonization in dyads from LMICs, although with significant heterogeneity in sampling methods. From MDR-GN dyads, molecular methods rarely found relatedness. Many studies suggested horizontal transmission within the environment.

**Conclusions:**

In LMICs, maternal MDR-GN colonization rarely results in vertical transmission to neonates. However, literature remains scarce and further research is needed.

## Teaser

This review summarizes literature from low- and middle-income countries detailing molecular evidence of vertical transmission as a cause of neonatal multidrug-resistant gram-negative colonization. Contrary to findings in high-income settings, vertical transmission of multidrug-resistant gram-negative colonization appears rare in low- and middle-income countries.

In low- and middle-income countries (LMICs), neonatal sepsis from multidrug-resistant gram-negative (MDR-GN) bacteria is a leading cause of under-5 mortality [1, 2]. While access to neonatal intensive care services is essential to advancing the care of premature and low birth weight infants, high MDR-GN transmission rates in LMIC neonatal intensive care units threaten efforts to provide effective and timely antimicrobial therapy for neonatal sepsis. Facing rising rates of global antimicrobial resistance, the World Health Organization has identified investigation of factors driving neonatal infections by resistant pathogens as an area of research priority [3]. As neonatal MDR-GN colonization often precedes invasive infection, addressing risk factors for neonatal MDR-GN colonization is essential to reduce neonatal sepsis-related mortality [4].

When causes of neonatal MDR-GN colonization have been explored, maternal colonization with antibiotic-resistant bacteria has historically been viewed as an important risk factor [5–7]. In many LMIC settings, community MDR-GN colonization prevalence ranges from 16.7% to 74.5% [8], leading to substantial detection of maternal MDR-GN colonization at the time of delivery [9, 10]. However, as MDR-GN colonization encompasses a broad range of pathogens, comparing rates of maternal and neonatal MDR-GN colonization does not adequately account for the nuances of maternal and neonatal colonization with different gram-negative species. When detailed further, mothers from LMICs are most frequently colonized with *Escherichia coli*, while infants are colonized with *Klebsiella pneumoniae* [11–13]. In this context, MDR-GN colonization would not represent vertical transmission but rather neonatal acquisition driven by selective antibiotic pressure by selective antibiotic pressure [14–18]. Data framing maternal MDR-GN colonization as a strong risk factor for neonatal colonization have informed infection prevention and control (IPC) policies, which may discourage maternal skin-to-skin contact via kangaroo mother care (KMC) [19]. Yet, the practice of extended maternal skin-to-skin contact via KMC is considered an integral part of newborn care in LMICs and has been shown to significantly decrease neonatal sepsis-related mortality [20]. Therefore, recommendations for isolation procedures in the setting of maternal MDR-GN colonization must be weighed against the protective benefits of KMC in LMIC contexts.

Molecular techniques such as whole genome sequencing have become a cornerstone of IPC efforts to evaluate pathogen transmission and control outbreaks in real time [21–23]. When mothers and infants are colonized with the same species of MDR-GN, molecular detection methods can provide a targeted assessment of isolate relatedness, suggesting presumed vertical transmission, at the strain level [24, 25]. One systematic review and meta-analysis from 2020 evaluated molecular evidence of MDR-GN relatedness between mothers and their infants and found a 27% pooled prevalence of MDR-GN vertical transmission [26]. While this meta-analysis concluded that maternal MDR-GN colonization is strongly correlated with neonatal colonization, it is notable that all 6 studies in the meta-analysis were from upper middle– and high-income countries, with no data on this effect in LMICs. In the last 5 years, new data have emerged regarding molecular evaluation of neonatal and maternal MDR-GN colonization in LMICs [27–29]. Thus, the objective of our study was to systematically review English-language literature from LMICs to assess molecular relatedness between maternal and neonatal MDR-GN colonization isolates to characterize the extent of vertical transmission in these settings.

## METHODS

### Study Design

This study was a systematic review of English literature evaluating molecular evidence for the relatedness of MDR-GN organisms from colonized neonates and their mothers in LMICs. The systematic review was performed following PRISMA guidelines (Preferred Reporting Items for Systematic Reviews and Meta-analyses) [30]. See Appendix 1 for full information regarding the PRISMA checklist.

### Search Strategy

**Figure 1.**
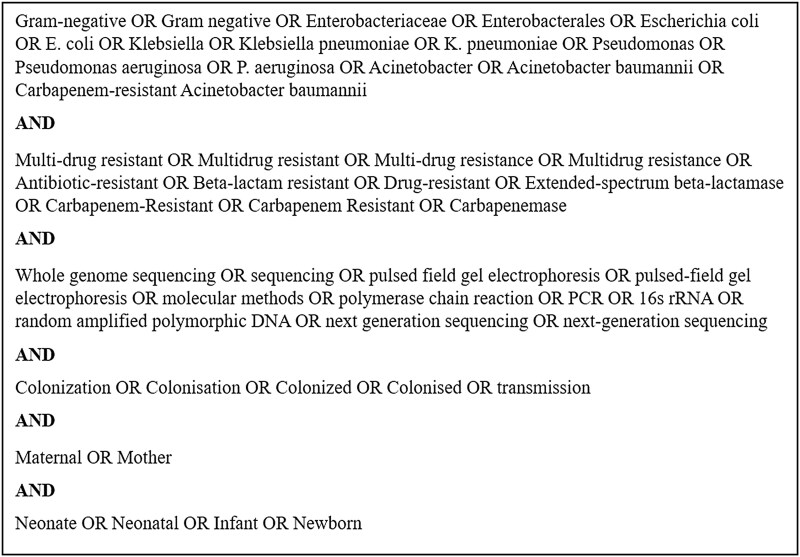
Database search strategy.

The search strategy was agreed on by all authors and modeled from similar reviews [26]. PubMed and Scopus databases were searched for primary literature that reported molecular evidence of MDR-GN resistance patterns for neonates and their mothers. MDR-GN pathogens of significance were identified as Enterobacterales (ie, *E coli* and *Klebsiella* species), *Pseudomonas aeruginosa*, and carbapenem-resistant *Acinetobacter baumannii* [31]. Accepted molecular detection techniques for strain-level identification included pulsed-field gel electrophoresis and polymerase chain reaction–based detection methods, such as 16-second rRNA sequencing, random amplified polymorphic DNA sequencing, next-generation sequencing, and whole genome sequencing [32]. The search was structured to include different iterations of descriptive terminology for antibiotic-resistant gram-negative organisms and terms referring to maternal and neonatal colonization. The search was performed on 29 May 2025. For the full search strategy, see Figure 1.

### Eligibility Criteria

Electronic databases were searched from their inception to 29 May 2025, and no date restrictions were applied. Articles were included if they were published in English and if the studied population was from an LMIC at the time of publication, as defined by the World Bank [33]. Studies were included if they reported molecular evidence of relatedness for MDR-GN colonization isolates from neonates and their mothers. Primary observational studies of any design, retrospective and prospective, were considered.

Studies were excluded if they did not include an evaluation of paired maternal-neonatal samples. Studies were also excluded if they discussed the relatedness of maternal and neonatal MDR-GN colonization but did not provide molecular evidence for comparison of individual isolates. Studies evaluating outbreak clusters were also excluded, as we deemed that outbreak scenarios would not accurately represent typical transmission patterns between mothers and neonates. Case reports or case series were not considered.

### Definitions

#### Multidrug Resistance in Gram-Negative Bacteria

Multidrug resistance was defined in concordance with international consensus criteria as nonsusceptibility to ≥1 antibiotic in ≥3 antibiotic groups, with the following antibiotic groups used in the classification: penicillins, penicillin + B-lactamase inhibitors (amoxicillin-clavulanic acid), antipseudomonal penicillins + B-lactamase inhibitors (piperacillin-tazobactam), aminoglycosides (gentamicin, amikacin), fluoroquinolones (ciprofloxacin, levofloxacin), folate pathway inhibitors (trimethoprim-sulfamethoxazole), extended-spectrum cephalosporins (ceftriaxone, ceftazidime, and cefepime), and carbapenems [34]. We also considered significant the detection of specific genotypic resistance mechanisms such as the production of extended-spectrum beta-lactamases (ESBLs; ESBL-Enterobacteriaceae) or carbapenemases (specifically carbapenem-resistant Enterobacteriaceae [CRE]).

#### Colonization Status

Colonization status was defined as isolation of organisms from the internal surfaces of a host (eg, gastrointestinal or genital tract) or the external (eg, skin, nares) without clinical evidence of disease [35].

### Selection Process

**Figure 2.**
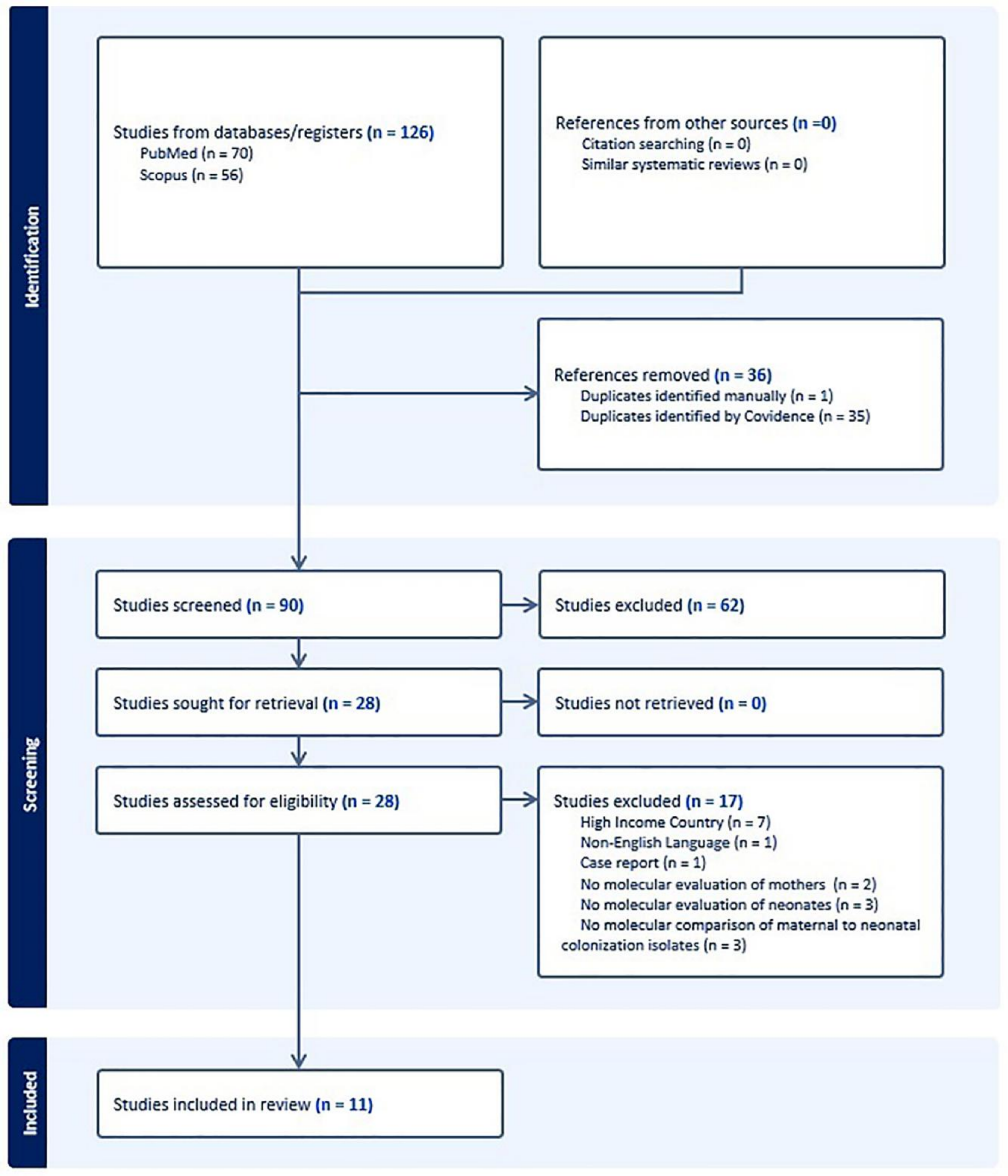
PRISMA flowchart.

The initial search resulted in 90 articles. All articles were uploaded to COVIDence, an online platform that allows for streamlining of the systematic review process [36]. Duplicate articles were automatically removed upon upload to the COVIDence system. G. S. and G. O.-K. independently reviewed each abstract for eligibility criteria, and those found to meet inclusion criteria were marked for full-text review. Differences in opinion of eligibility at this stage were resolved by another reviewer (J. A. D.) prior to moving forward. In the next stage, G. S. and G. O.-K. independently reviewed all full-text articles to ensure eligibility for inclusion, and differences were again resolved by J. A. D. Reference lists for each full-text article and related systematic reviews [11, 26, 37, 38] were manually reviewed by G. S., although this did not yield any additional articles for inclusion. Figure 2 details the PRISMA flowchart of the systematic review.

### Quality Assessment

All full-text articles underwent quality assessment with use of the Joanna Briggs Institute (JBI) critical appraisal tool for analytic cross-sectional or cohort studies as appropriate [39, 40]. Each JBI checklist reflects specific quality criteria relevant to the study design, with greater inclusion of quality measures indicating lower risk of bias. Items on the checklist are scored 1 = yes, 0 = no/unclear, or N/A = not applicable. Modeled from cutoffs established by prior systematic reviews, studies including >70% of applicable items were considered good quality/low risk of bias, 50% to 69% as moderate quality/moderate risk of bias, and <50% as low quality/high risk of bias [41, 42]. Articles were assessed by the study design as it related to neonatal colonization outcomes; for example, if a study primarily followed a longitudinal cohort of mothers but obtained only 1 associated neonatal colonization sample, this was assessed as a cross-sectional study. Articles were reviewed independently by G. S. and G. O.-K., with conflicts in assigned scores resolved through discussion between the reviewers. All studies meeting eligibility criteria were included for analysis regardless of study quality, as these were still felt to provide important contributions to the review.

### Data Extraction

Data were independently extracted by G. S. and G. O.-K. from each report and uploaded into a REDCap database designed by N. L. Variables for extraction were predefined and fell into 4 broad categories: (1) study setting and population, (2) approach to colonization sampling and laboratory analysis of isolates, (3) distribution of neonatal and maternal gram-negative colonization and associated antibiotic resistance, and (4) molecular relatedness of resistant maternal and neonatal isolates. If evaluated, relatedness of maternal and neonatal isolates to environmental isolates was also included. All relevant data were extracted from published studies without identification of individual patients. When clarification was required, study authors were contacted to provide additional information. However, by the time of manuscript submission, the 2 study authors contacted had not responded to request for further data.

### Statistical Analysis

To estimate the prevalence of MDR-GN, the total number of MDR-GN cases and number of participants, maternal and infant, were extracted. If >1 report of total number of participants in any study was provided, the participant count at the latest date of sampling was extracted for prevalence estimation. The proportion of participants colonized with MDR was calculated for each study and each participant type, and 95% CI estimates of the population prevalence were calculated via Clopper-Pearson methods. Forest plots for each MDR-GN type (ESBL, CRE, MDR) were constructed to graphically compare the proportion of participants colonized with MDR-GN. Due to heterogeneity in study design, no pooled prevalence was calculated. Statistical analysis was performed with Stata-BE 18.0.

## RESULTS

From the 90 articles identified per the original search criteria, 11 full-text articles were included for data extraction and quality assessment (Table 1) [27–29, 43–50]. The studies were predominantly from countries in sub-Saharan Africa (n = 6) [28, 44, 46, 47, 49, 50] and Southeast Asia (n = 4) [29, 43, 45, 48], with 1 study conducted as a part of a multicountry cohort across 12 clinical sites in multiple LMICs [27]. Most studies were descriptive rather than analytic, providing prevalence assessments of MDR colonization in settings where this had not previously been described. While no date restrictions were applied, all included studies were published between 2018 and 2024, reflecting the rapidly evolving landscape of global antimicrobial resistance research in the last 10 years. Many studies assessed neonatal and maternal colonization status by performing rectal swabs around the time of delivery, although timing and collection method varied greatly. For example, Chomkatekaew et al obtained neonatal rectal swabs within 24 hours of hospital admission; however, due to a fully outborn population, this equated to sampling at an average of 11 days after birth [29]. Other studies collected only nasal, oral, or skin swabs from infants, leading to variation in prevalence assessments. For instance, Njeuna et al assessed neonatal MDR-GN colonization by collecting nasopharyngeal swabs from neonates, with lower prevalence (19/90, 21%) likely attributable to sampling an area with decreased yield for these enteric pathogens [50]. There was also notable heterogeneity in populations of interest: while most studies recruited all consecutive mothers presenting for obstetric care, Mayanja et al evaluated only mothers admitted for cesarean delivery [28]. Yet, in total, the studies reflect high rates of maternal and neonatal MDR colonization in LMIC settings. Most studies (n = 7) [27, 29, 44–47, 49] utilized whole genome sequencing for assessment of MDR-GN molecular relatedness between dyads, although other polymerase chain reaction–based methods and random amplified polymorphic DNA were included [28, 43, 48, 50].

**Table 1.** Molecular Relatedness of Maternal and Neonatal MDR-GN Colonization Isolates in LMICs

Publication	Study Design, Duration, and Setting	Population and No. of Mother-Neonate Dyads	Outcome of Interest	Source and Timing of Colonization Sampling	Molecular Methods and Definition of Isolate Relatedness	Colonization Outcomes and Relatedness of Isolates From Paired Dyads	Evidence of Isolate Relatedness to Environmental Samples or Other Study Participants
Nanayakkara (2018) [43]	Prospective cohort study in overarching design; cross-sectional from the perspective of neonatal colonization sampling. Oct 2015–Jan 2016. Obstetric ward at a referral hospital in Sri Lanka.	Women admitted for term vaginal delivery and their liveborn infants. Excluded women who had heavy vaginal bleeding or underwent emergency C-section and dyads where the neonate was admitted to the special baby care unit. 159 dyads enrolled.	Colonization with select neonatal bacterial pathogens (*S agalactiae*, *Enterococcus* spp, *E coli*, *Klebsiella* spp) with attention to MDR phenotypes.	Neonatal perirectal swabs were obtained at the time of discharge. Maternal vaginal swabs were obtained at the time of admission and again upon discharge.	Random amplification of polymorphic DNA. Isolates considered related if they demonstrated similarity >75% in the Dice index at a tolerance of 3.5.	54/159 (34%) neonates were colonized with Enterobacteriaceae, with 2/159 (1%) colonized with ESBL-E and no MDR colonization. 7/250 (2.8%) mothers were ESBL colonized and 1/250 (0.4%) MDR colonized Only 1 dyad was colonized with related ESBL-E isolates.	Not assessed
Rakotondrasoa (2020) [44]	Prospective cohort study. 2012–2016. Community centers and home visits in Madagascar.	Women presenting for third trimester antenatal visits and their liveborn infants. 2001 dyads enrolled.	*K pneumoniae*	Deep ear canal and anal swabs obtained from neonates at birth and then at routine follow-up as indicated until 18 mo of life. Rectal swabs or stool samples collected from mothers at third trimester antenatal visits.	WGS performed with calculation of SNP distance between isolates.	100/2001 (5%) infants and 101/2000 (5%) mothers demonstrated *K pneumoniae* colonization, with 65 isolates found to be MDR phenotype. Two dyads were colonized with genotypically related MDR *K pneumoniae*	Not assessed
Meredith (2021) [45]	Prospective cohort study. Aug 2016–Aug 2017. Obstetric ward at a referral hospital in Sri Lanka.	Women >18 y of age presenting in labor and their liveborn infants. Excluded women with active rectal bleeding or those who lived >40 km from the hospital. 199 dyads enrolled.	MDR-GN	Neonatal and maternal rectal swabs were obtained within 48 h of delivery and again 4–6 wk after discharge.	WGS performed with calculation of SNP distance between isolates. Only *E coli* isolates were sequenced.	27/199 (13.5%) infants were MDR-GN colonized: 3 at enrollment and 24 at posthospital reassessment. 50/199 (25%) mothers were MDR-GN colonized: 24 at enrollment and 26 at reassessment. Six dyads demonstrated relatedness of isolates.	Not assessed
Carvalho (2022) [27]	Prospective cohort study in overarching design; cross-sectional from the perspective of neonatal colonization sampling. Nov 2015–Nov 2017. Part of the multicountry BARNARDS study across 12 clinical sites in LMICs.	Recruited all mothers in labor at participating clinical sites and enrolled neonates after birth. Additionally recruited infants <60 d hospitalized with suspected sepsis. BARNARDS recruited 35 040 mothers and their 36 285 neonates; this study analyzed a subset of 15 217 maternal and 2931 neonatal samples.	ESBL-E, CRE	Neonatal rectal swabs collected as soon as possible after birth. Maternal rectal swabs collected upon enrollment.	Multiplex PCR performed for identification of relevant AMR genes (*bla*-CTXM, *bla*-NDM, *bla*-OXA48, and *bla*-KPC), with WGS performed on a subset of CRE from select sites in Nigeria, Bangladesh, and Pakistan. Relatedness determined by SNP distance.	1644/2931 (56%) neonates and 7167/15217 (47%) mothers were ESBL-E positive. For CRE, of 2931 neonates, 542 (18.5%) carried for *bla*-NDM, 120 (4.1%) *bla*-OXA48, with no *bla*-KPC detected. Of 15 217 mothers, 700 (4.6%) carried *bla*-NDM, 243 (1.6%) *bla*-OXA48, and 8 (0.05%) *bla*-KPC. 422 isolates underwent WGS with 4 episodes of related strains shared between dyads (1 *E coli*, 3 *K pneumoniae*).	The 1 *E coli* strain ST405 shared between a mother and neonate at a hospital in Bangladesh was shared with 7 other mothers and 4 other neonates (all within SNP distance of 6). A separate hospital in Bangladesh had 8 mothers and 5 neonates with the same strain of *E cloacae* ST68 (SNP distance, 4).
Villinger (2022) [46]	Prospective cohort study. Jan–Apr 2019. Neonatal ward at a referral hospital in Kenya.	Recruited all consecutive dyads admitted to the neonatal ward who provided informed consent. 300 dyads enrolled.	MDR bacteria with attention to CRE	Neonatal rectal swabs obtained on day of admission and weekly thereafter until discharge. Maternal vaginal swabs collected once upon enrollment.	WGS performed with calculation of SNP distance between isolates.	48/300 (16%) and 8/300 (2.6%) neonates were respectively MDR and CRE colonized at admission with 97/218 (44.5%) and 29/218 (13%) MDR and CRE colonized by discharge. 45/300 (15%) mothers were MDR colonized and 7/300 (2%) were CRE colonized. Only 3 dyads had colonization with the same species (all MDR *K pneumoniae*), but none were genetically related (SNP distances all >10 000).	Twenty clusters of related MDR bacteria were shared among neonates, mothers, and the environment, with 1 example of ESBL ST39 *K pneumoniae* colonizing 15 neonates over the study period.
Bah (2023) [47]	Prospective cohort study. Apr–Aug 2017. Neonatal ward at a referral hospital in The Gambia.	All neonates born at the hospital weighing <2 kg and aged <24 h and their mothers. Excluded dyads with gastrointestinal pathologies or maternal HIV or other STI or if neonatal death occurred before or after screening. 34 neonates enrolled with 21 mother-neonate pairs.	MDR-GN, ESBL-E	Neonatal skin and perirectal swabs were obtained as soon as possible after admission and weekly until discharge, death, or 28 d of life. Maternal rectovaginal swabs obtained once within 72 h of neonatal admission.	WGS performed with calculation of SNP distance between isolates.	14/34 (41%) neonates were MDR/ESBL colonized at admission, with 13/13 (100%) remaining neonates colonized by day 7 of hospitalization. 16/21 (76%) mothers were MDR/ESBL colonized. Only 1 dyad had transmission of genetically identical (SNP distance, 0) *K pneumoniae and E coli*, which were both isolated from the neonate within 24 h of admission.	One set of neonate twins carried identical *K pneumoniae* (SNP distance, 0), which was not identified in their mother.
Chomkatekaew (2023) [29]	Cross-sectional study. Mar 2016–Mar 2017. Neonatal wards at a referral hospital in Cambodia.	All infants <28 d upon admission. Fully outborn population excluded infants with prior overnight admission to a health center. 50 dyads enrolled.	ESBl-E	Neonatal rectal swabs obtained within 24 h of admission, at a median age of 11 d. Maternal rectal swabs were collected as soon as possible after infant admission.	WGS performed with calculation of SNP distance between isolates.	25/50 (50%) neonates and 47/50% mothers were colonized with phenotypic ESBL-E, with high rates of MDR confirmed by genotypic sequencing. Out of 21 dyads colonized with the same organism, 8 were genotypically related.	One example of a related *E coli* pair (SNP distance, 13) from an unpaired mother and neonate collected 54 d apart.
Mayanja (2023) [28]	Cross-sectional study. Aug 2015–Aug 2016. Obstetric ward at a referral hospital in Uganda.	Mothers admitted for cesarean delivery and their liveborn infants. 137 dyads enrolled.	Enterobacteriaciae colonization with attention to ESBL-E and CRE	Neonatal and maternal swabs from nasal, axillary, and inguinal sources collected at birth and for neonates again upon discharge.	PCR-based detection of identical antibiotic resistance profiles for spatial cluster analysis of genotypic relatedness	14/137 (10%) neonates and 16/137 (11.6%) mothers were MDR-GN colonized. Two dyads had identical isolates based on molecular detection of AMR profiles.	Eight transmission clusters involving mothers, neonates, health care workers, and environmental surfaces were detected.
Dutta (2024) [48]	Prospective cohort study. Dec 2018–Aug 2020. Obstetric and neonatal wards at a referral hospital in India.	Preterm breastfeeding infants (gestation, 28–34 wk) and their mothers. Excluded dyads where infants had gastrointestinal surgical concerns, life expectancy <7 d, or mothers with mastitis. 100 dyads enrolled.	Presence of 13 potential neonatal pathogens, including several gram-negative bacteria (*E coli*, *K pneumoniae*, *E cloacae*, *P aeruginosa*, *A baumannii*) with attention to MDR phenotypes	Neonatal oral and rectal swabs obtained at enrollment and weekly thereafter. Samples of maternal expressed breastmilk were collected once under sterile procedure by 2 research fellows.	Multilocus sequence typing performed with 7 housekeeping genes to determine concordance in these genes between mother-neonate pairs.	Overall, 80/100 (80%) neonates and 9/100 (9%) mothers were colonized with pathogenic MDR organisms. Only 1 dyad had genetically identical MDR *E coli* (ST405).	Not assessed
Dos Santos (2024) [49]	Cross-sectional study. Feb–June 2022. Obstetric and neonatal wards at a referral hospital in Gabon.	Recruited all consecutive mothers presenting in labor and their infants after birth. 203 dyads enrolled.	CRE	Neonatal rectal swabs obtained within 30 min of birth. Maternal rectal swabs obtained immediately before delivery.	WGS performed with calculation of SNP distance between isolates.	12/203 (5.9%) mothers were CRE colonized. Only 1/203 (0.5%) neonate was colonized with CRE. This isolate was a ST45 *K pneumoniae* that expressed phenotypic carbapenem resistance without harboring a carbapenemase gene. This isolate was shared with the neonate's mother. Notably, 1 infant developed CRE bacteremia during the study, whose mother was not a CRE carrier.	Multiple related CRE isolates detected between mothers and related environmental samples, including ST410 *E coli* shared by 4 mothers and a sink.
Njeuna (2024) [50]	Cross-sectional study. Feb–June 2022. Delivery hospital in Cameroon.	Women presenting in labor at gestation >32 wk and their liveborn infants. Excluded if mothers had mental health concerns or neonates had a poor health prognosis. 90 dyads enrolled.	*E coli* and *K pneumoniae* with attention to ESBL and MDR phenotypes.	Nasopharyngeal swabs collected from neonates within 24 h of life. Maternal rectovaginal swabs obtained prior to delivery.	Enterobacterial repetitive intergenic consensus–PCR with comparison of genomic fingerprints	19/90 (21%) neonates and 73/93 (78%) mothers were positive for MDR *E coli* or *K pneumoniae*. Only 1 dyad had a pair of *E coli* isolates with 100% similarity, though displayed discordant MDR phenotype.	The mother-neonate pair of related *E coli* isolates shared common ancestors with 1 mother and 2 neonates collected 7 d prior. A pair of isolates with 100% similarity was shared between a neonate and a different mother, which shared common ancestors with isolates from a health care worker.

Abbreviations: AMR, antimicrobial resistance; CRE, carbapenem-resistant Enterobacteriaceae; ESBL, extended-spectrum beta-lactamase; ESBL-E, extended-spectrum beta-lactamase–Enterobacteriaceae; GN, gram negative; LMICs, low- and middle-income countries; MDR, multidrug resistant; PCR, polymerase chain reaction; SNP, single-nucleotide polymorphism; STI, sexually transmitted infection; WGS, whole genome sequencing.

**Figure 3.**
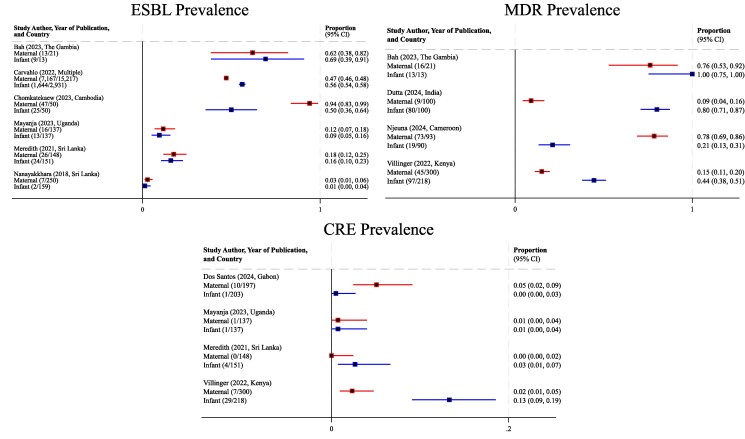
Forest plots comparing maternal and infant colonization proportions with prevalence estimates (95% CIs). *A*, Significant heterogeneity was noted across studies; therefore, no pooled estimates were calculated. *B*, Prevalence proportions were unable to be calculated from Rakotondrasoa et al [44], as reports of MDR gram-negative prevalence did not differentiate between maternal and neonatal isolates. CRE, carbapenem-resistant Enterobacteriaceae; ESBL, extended-spectrum beta-lactamase; MDR, multidrug resistant.

The estimated gram-negative colonization prevalences varied significantly among study types and between mothers and infants (Figure 3). Some studies reported a similar proportion of mother and infant colonization with MDR-GN; however, many showed dissimilarity between maternal and infant colonization rates. Nevertheless, all studies were alike in demonstrating infrequent evidence of related colonization isolates, irrespective of overall prevalence for mothers and neonates. Furthermore, many studies found evidence of horizontal transmission within the hospital environment between mothers, between neonates, and between mothers with unrelated neonates [27–29, 46, 47, 49, 50]. Most notably, Dos Santos et al reported an instance of ST410 *E coli* shared by 4 mothers and a communal sink, demonstrating that environmental surfaces may serve as sources of acquisition for not only neonates but also mothers staying in the hospital [49].

Regarding quality assessment, 8 articles were classified as good quality with low risk of bias [27, 29, 43–46, 49, 50] and 3 articles as moderate quality with moderate risk of bias [28, 47, 48]. The quality assessment scoring based on JBI criteria can be found in Appendix 2. Studies of moderate quality infrequently provided assessment of potential confounding variables, although in several cases this was not relevant given the descriptive rather than analytic nature of the study. For cohort studies, it was not possible to determine if participants were free of the outcome (neonatal MDR-GN colonization) at the time of exposure (maternal MDR-GN colonization), although all studies provided valid methods of measurement for exposures and outcomes.

## DISCUSSION

From this systematic review, we identified 11 studies detailing the molecular relatedness of maternal and neonatal MDR-GN colonization isolates from LMIC contexts. The limited number of studies published on this topic is notable, indicating the need for further high-quality research on routes of neonatal MDR-GN acquisition in LMICs. Findings from this review demonstrate a high prevalence of neonatal MDR-GN colonization ranging from 21% to 100%, although evidence of vertical transmission by molecular methods was rarely detected in any study. This contrasts with the prior review from high-income countries that implicated maternal MDR-GN colonization as a significant risk factor for neonatal MDR-GN colonization [26].

The studies in this review varied widely in estimations of MDR-GN colonization prevalence; however, similar literature describes alarming rates of neonatal MDR-GN colonization in LMICs. In a 2024 systematic review on neonatal colonization with antibiotic-resistant pathogens in LMICs, Beaumont et al reported a pooled prevalence estimate of 30.2% for neonatal third-generation cephalosporin-resistant Enterobacterales colonization and 2.6% for CRE colonization [11]. While there was a significant association between prolonged rupture of membranes and neonatal third-generation cephalosporin-resistant Enterobacterales colonization, the review did not specifically evaluate the impact of maternal MDR-GN colonization on neonatal MDR-GN colonization. The authors suggest that this association may not be solely due to vertical transmission and could instead reflect the fact that neonates with prolonged rupture of membranes may require more intensive resuscitation measures and greater exposure to hospital reservoirs of MDR-GN organisms. Our review is the first to evaluate molecular evidence of the role of vertical transmission on neonatal MDR-GN colonization in LMICs, with consistent examples of unrelated neonatal and maternal isolates. Our findings suggest that in LMICs, it is unlikely that mothers serve as the most significant reservoir of neonatal MDR-GN colonization. Furthermore, many mothers had shared isolates in the context of a shared environment, raising concern that dyadic concordance could be due to combined horizontal acquisition rather than vertical transmission in a setting with high MDR-GN prevalence. This review highlights the need for ongoing studies on this topic to elucidate the role of vertical and horizontal transmission of MDR-GN in LMICs, which will inform IPC policies to prevent neonatal sepsis.

While colonization outcomes related to neonatal sepsis were not evaluated in this review, our conclusions are supported by 1 genomic analysis study detailing low rates of vertical transmission leading to neonatal sepsis in The Gambia [51]. From 203 enrolled dyads with early-onset neonatal sepsis where maternal colonization swabs were obtained, 36 neonates had clinically significant bacteremia, but no gram-negative organisms were genetically related to maternal colonization isolates. This concept is supported by a large randomized controlled trial demonstrating that intrapartum vaginal cleansing with chlorhexidine gluconate did not result in a lowered incidence of neonatal sepsis, indicating that a reduction in maternal colonization burden does not reduce neonatal acquisition of pathogenic bacteria [52]. Similarly, the well-established role of maternal group B *Streptococcus* colonization in early-onset neonatal sepsis underscores the importance of thoroughly characterizing reservoirs and transmission pathways for MDR-GN organisms, even though such transmission was rarely demonstrated in the studies reviewed. Further evaluation of this relationship between maternal colonization status and neonatal sepsis outcomes is critical for framing the utilization of maternal skin-to-skin contact via KMC and its role in IPC practices. A growing body of evidence supports KMC as an effective measure to reduce neonatal sepsis-related mortality in LMICs, leading the World Health Organization to recommend initiation of KMC for low birth weight infants as soon as they are clinically stable [53]. The method by which KMC reduces neonatal sepsis-related mortality remains unclear; however, it is possible that early exposure to the maternal microbiome could offer protection against acquisition of pathogenic bacteria from the hospital environment [54, 55]. Notably, 1 study from Estonia, a high-income country, found that early introduction of family integrated care inclusive of KMC resulted in improved neonatal microbiome diversity and reduced colonization with hospital-acquired strains of Enterobacteriaceae, although these findings have yet to be replicated in an LMIC environment [56]. Based on the available limited evidence, the findings of this systematic review support the notion that in LMICs, maternal MDR-GN colonization does not bestow undue risk to the neonate and should not lead to isolation practices that discourage KMC. Given standard practices where premature and low birth weight infants are separated from their mothers for initial stabilization, further research is needed to understand whether implementation of early KMC can serve as a sustainable IPC measure against neonatal MDR-GN acquisition and thereby sepsis in LMICs. While this review did capture studies evaluating several sampling time points within the neonatal period, the scarcity of available data identified by this systematic review highlights the need for additional investigation into the timing of mother-to-child MDR-GN transmission (ie, perinatal vs postnatal) to identify potential targets for IPC interventions in LMICs. Future research efforts should also aim to balance strategies that will be broadly applicable in LMICs by identifying targeted approaches unique to local contexts with regard to specific antimicrobial resistance challenges and available resources.

### Limitations

This study has several limitations, primarily the small number of studies based on a lack of published literature on this topic. This review excluded non-English literature, and while only 1 study from the queried databases was excluded for this reason, it remains possible other non-English publications from LMICs may have been missed. The quality of the studies varied, although notably none of the studies were of poor quality. The review did not include literature implying dyadic concordance based on antimicrobial susceptibility profiles alone; while this would lead to exclusion of studies that did not have the funding or capability to perform molecular analysis in a resource-limited setting, this was a deliberate choice to focus only on molecular analysis as the most accurate representation of true relatedness. Diversity in study design, such as location and timing of sample collection, whether samples were obtained once or longitudinally, and mixed inborn-outborn populations, may limit generalizability of these findings. There is also the issue of within-host colonization diversity and colony selection bias, where molecular evaluation of only 1 strain per participant could mask true relatedness of other colonizing strains between dyads.

While many adjacent systematic reviews have performed meta-analysis on vertical transmission data, we determined that this would risk providing an inaccurate assessment of the true incidence rate in LMIC contexts. This decision was due to significant variation in timing of colonization sampling and outcomes of interest. For example, Dos Santos et al obtained neonatal rectal swabs within 30 minutes of birth to evaluate CRE prevalence [49], while Chomkatekaew et al collected neonatal rectal colonization swabs at a median 11 days of life to evaluate the prevalence of ESBL-Enterobacteriaceae [29]. Variation in colonization rates based on timing of sampling likely reflects differential exposure to hospital reservoirs: Dos Santos et al found low rates of neonatal CRE colonization in the context of early neonatal sampling, while Villinger et al found higher neonatal CRE colonization after longitudinal exposure to the neonatal intensive care unit environment [46]. There were also challenges with interpreting the quality of data, including many examples where a significant number of samples were unable to be recovered for sequencing purposes. From this, it was deemed that distilling these heterogenous studies down to a discrete proportion of MDR-GN vertical transmission would risk misrepresenting the data and would not be appropriately corrected by a random effects model. Together, these limitations demonstrate the need for high-quality publications on evidence of MDR-GN vertical transmissions in LMIC settings.

## CONCLUSION

In summary, while neonates and their mothers from LMICs experience high rates of MDR-GN colonization, there is limited molecular evidence of vertical transmission. Efforts to detail pathways of neonatal MDR-GN acquisition in LMICs should be prioritized to effectively inform IPC interventions and reduce the rising burden of neonatal MDR-GN sepsis.

## Supplementary Material

ofag010_Supplementary_Data

## Supplementary Data

Supplementary materials are available at *Open Forum Infectious Diseases* online. Consisting of data provided by the authors to benefit the reader, the posted materials are not copyedited and are the sole responsibility of the authors, so questions or comments should be addressed to the corresponding author.

## References

[1] Rudd KE, Johnson SC, Agesa KM, et al Global, regional, and national sepsis incidence and mortality, 1990–2017: analysis for the Global Burden of Disease Study. Lancet 2020; 395:200–11. 31954465 10.1016/S0140-6736(19)32989-7 PMC6970225

[2] Flannery DD, Chiotos K, Gerber JS, Puopolo KM. Neonatal multidrug-resistant gram-negative infection: epidemiology, mechanisms of resistance, and management. Pediatr Res 2022; 91:380–91. 34599280 10.1038/s41390-021-01745-7 PMC8819496

[3] World Health Organization . Global research agenda for antimicrobial resistance in human health policy brief. Available at: https://cdn.who.int/media/docs/default-source/antimicrobial-resistance/amr-spc-npm/who-global-research-agenda-for-amr-in-human-health—policy-brief.pdf?sfvrsn=f86aa073_4&download=true. Accessed 22 June 2025.

[4] Folgori L, Tersigni C, Hsia Y, et al The relationship between gram-negative colonization and bloodstream infections in neonates: a systematic review and meta-analysis. Clin Microbiol Infect 2018; 24:251–7. 28830807 10.1016/j.cmi.2017.08.008

[5] Denkel LA, Schwab F, Kola A, et al The mother as most important risk factor for colonization of very low birth weight (VLBW) infants with extended-spectrum β-lactamase–producing Enterobacteriaceae (ESBL-E). J Antimicrob Chemother 2014; 69:2230–7. 24729603 10.1093/jac/dku097

[6] Patangia DV, Ryan CA, Dempsey E, Stanton C, Ross RP. Vertical transfer of antibiotics and antibiotic resistant strains across the mother/baby axis. Trends Microbiol 2022; 30:47–56. 34172345 10.1016/j.tim.2021.05.006

[7] Mady EA, Doghish AS, El-Dakroury WA, et al Impact of the mother's gut microbiota on infant microbiome and brain development. Neurosci Biobehav Rev 2023; 150:105195. 37100161 10.1016/j.neubiorev.2023.105195

[8] Bezabih YM, Bezabih A, Dion M, et al Comparison of the global prevalence and trend of human intestinal carriage of ESBL-producing *Escherichia coli* between healthcare and community settings: a systematic review and meta-analysis. JAC Antimicrob Resist 2022; 4:dlac048. 10.1093/jacamr/dlac048 PMC9160884 35668909

[9] Amsalu G, Wen CT, Perovic O, et al Carriage of antimicrobial-resistant Enterobacterales among pregnant women and newborns in Amhara, Ethiopia. Int J Infect Dis 2024; 143:107035. 38561043 10.1016/j.ijid.2024.107035 PMC11068590

[10] Ghaddar N, Anastasiadis E, Halimeh R, et al Phenotypic and genotypic characterization of extended-spectrum beta-lactamases produced by *Escherichia coli* colonizing pregnant women. Infect Dis Obstet Gynecol 2020; 2020:1–7. 10.1155/2020/4190306 PMC7168714 32327921

[11] Beaumont AL, Kermorvant-Duchemin E, Breurec S, Huynh BT. Neonatal colonization with antibiotic-resistant pathogens in low- and middle-income countries: a systematic review and meta-analysis. JAMA Netw Open 2024; 7:e2441596. 39499519 10.1001/jamanetworkopen.2024.41596 PMC11581591

[12] Zelellw DA, Dessie G, Worku Mengesha E, Balew Shiferaw M, Mela Merhaba M, Emishaw S. A systemic review and meta-analysis of the leading pathogens causing neonatal sepsis in developing countries. Biomed Res Int 2021; 2021:6626983. 34195273 10.1155/2021/6626983 PMC8203353

[13] Sakai AM, Iensue TNAN, Pereira KO, et al Colonization by multidrug-resistant microorganisms of hospitalized newborns and their mothers in the neonatal unit context. J Infect Dev Ctries 2020; 14:765–71. 32794468 10.3855/jidc.12091

[14] Dramowski A, Aucamp M, Beales E, et al Healthcare-associated infection prevention interventions for neonates in resource-limited settings. Front Pediatr 2022; 10: 919403. 35874586 10.3389/fped.2022.919403 PMC9301049

[15] Turner P, Pol S, Soeng S, et al High prevalence of antimicrobial-resistant gram-negative colonization in hospitalized Cambodian infants. Pediatr Infect Dis J 2016; 35:856–61. 27124686 10.1097/INF.0000000000001187 PMC4957964

[16] Diorio-Toth L, Wallace MA, Farnsworth CW, et al Intensive care unit sinks are persistently colonized with multidrug resistant bacteria and mobilizable, resistance-conferring plasmids. mSystems 2023; 8:e0020623. 37439570 10.1128/msystems.00206-23 PMC10469867

[17] Vurayai M, Strysko J, Kgomanyane K, et al Characterizing the bioburden of ESBL-producing organisms in a neonatal unit using chromogenic culture media: a feasible and efficient environmental sampling method. Antimicrob Resist Infect Control 2022; 11:14. 35074019 10.1186/s13756-021-01042-2 PMC8785036

[18] Marando R, Seni J, Mirambo MM, et al Predictors of the extended-spectrum-beta lactamases producing Enterobacteriaceae neonatal sepsis at a tertiary hospital, Tanzania. Int J Med Microbiol 2018; 308:803–11. 29980372 10.1016/j.ijmm.2018.06.012 PMC6171784

[19] Johnson J, Quach C. Outbreaks in the neonatal ICU: a review of the literature. Curr Opin Infect Dis 2017; 30:395–403. 28582313 10.1097/QCO.0000000000000383 PMC8020806

[20] Arya S, Chhabra S, Singhal R, et al Effect on neonatal sepsis following immediate kangaroo mother care in a newborn intensive care unit: a post-hoc analysis of a multicentre, open-label, randomised controlled trial. EClinicalMedicine 2023; 60:102006. 37251633 10.1016/j.eclinm.2023.102006 PMC10209186

[21] Aanensen DM, Feil EJ, Holden MT, et al Whole-genome sequencing for routine pathogen surveillance in public health: a population snapshot of invasive *Staphylococcus aureus* in Europe. mBio 2016; 7:e00444-16. 27150362 10.1128/mBio.00444-16 PMC4959656

[22] Zhou K, Lokate M, Deurenberg RH, et al Use of whole-genome sequencing to trace, control and characterize the regional expansion of extended-spectrum β-lactamase producing ST15 *Klebsiella pneumoniae*. Sci Rep 2016; 6:20840. 26864946 10.1038/srep20840 PMC4749987

[23] Mutters NT, Heeg K, Späth I, Henny N, Günther F. Improvement of infection control management by routine molecular evaluation of pathogen clusters. Diagn Microbiol Infect Dis 2017; 88:82–7. 28189284 10.1016/j.diagmicrobio.2017.01.013

[24] Feehily C, O’Neill IJ, Walsh CJ, et al Detailed mapping of *Bifidobacterium* strain transmission from mother to infant via a dual culture-based and metagenomic approach. Nat Commun 2023; 14:3015. 37230981 10.1038/s41467-023-38694-0 PMC10213049

[25] Bogaert D, van Beveren GJ, de Koff EM, et al Mother-to-infant microbiota transmission and infant microbiota development across multiple body sites. Cell Host Microbe 2023; 31:447–60.e6. 36893737 10.1016/j.chom.2023.01.018

[26] Bulabula ANH, Dramowski A, Mehtar S. Transmission of multidrug-resistant gram-negative bacteria from colonized mothers to their infants: a systematic review and meta-analysis. J Hosp Infect 2020; 104:57–67. 31604126 10.1016/j.jhin.2019.10.001

[27] Carvalho MJ, Sands K, Thomson K, et al Antibiotic resistance genes in the gut microbiota of mothers and linked neonates with or without sepsis from low- and middle-income countries. Nat Microbiol 2022; 7:1337–47. 35927336 10.1038/s41564-022-01184-y PMC9417982

[28] Mayanja R, Muwonge A, Aruhomukama D, et al Source-tracking ESBL-producing bacteria at the maternity ward of Mulago hospital, Uganda. PLoS One 2023; 18:e0286955. 37289837 10.1371/journal.pone.0286955 PMC10249850

[29] Chomkatekaew C, Thaipadungpanit J, Hearn P, et al Detection of maternal transmission of resistant gram-negative bacteria in a Cambodian hospital setting. Front Microbiol 2023; 14:1158056. 37125167 10.3389/fmicb.2023.1158056 PMC10140293

[30] PRISMA . PRISMA: transparent reporting of systematic reviews and meta-analyses. Available at: https://www.prisma-statement.org/?AspxAutoDetectCookieSupport=1. Accessed 15 January 2025.

[31] Macesic N, Uhlemann AC, Peleg AY. Multidrug-resistant gram-negative bacterial infections. Lancet 2025; 405:257–72. 39826970 10.1016/S0140-6736(24)02081-6

[32] Gerace E, Mancuso G, Midiri A, Poidomani S, Zummo S, Biondo C. Recent advances in the use of molecular methods for the diagnosis of bacterial infections. Pathogens 2022; 11:663. 35745518 10.3390/pathogens11060663 PMC9229729

[33] World Bank . Low and middle income countries. Available at: https://data.worldbank.org/country/XO. Accessed 29 May 2025.

[34] Magiorakos AP, Srinivasan A, Carey RB, et al Multidrug-resistant, extensively drug-resistant and pandrug-resistant bacteria: an international expert proposal for interim standard definitions for acquired resistance. Clin Microbiol Infect 2012; 18:268–81. 21793988 10.1111/j.1469-0691.2011.03570.x

[35] Wohrley JD, Bartlett AH. The role of the environment and colonization in healthcare-associated infections. Healthcare-Associated Infections in Children 2018; 16:17–36.

[36] Veritas Health Innovation. Covidence systematic review software. Available at: http://www.covidence.org.

[37] Chan GJ, Lee AC, Baqui AH, Tan J, Black RE. Prevalence of early-onset neonatal infection among newborns of mothers with bacterial infection or colonization: a systematic review and meta-analysis. BMC Infect Dis 2015; 15:118. 25886298 10.1186/s12879-015-0813-3 PMC4364328

[38] Chan GJ, Lee AC, Baqui AH, Tan J, Black RE. Risk of early-onset neonatal infection with maternal infection or colonization: a global systematic review and meta-analysis. PLoS Med 2013; 10:e1001502. 23976885 10.1371/journal.pmed.1001502 PMC3747995

[39] Joanna Briggs Institute . Checklist for analytical cross sectional studies. 2017. Available at: https://jbi.global/critical-appraisal-tools. Accessed 31 July 2025.

[40] Joanna Briggs Institute . Checklist for cohort studies. 2017. Available at: https://jbi.global/critical-appraisal-tools. Accessed 31 July 2025.

[41] Franco A, Tereza M, Navarro M, Santos J, Silva RF, Paranhos LR. Evidence-based mapping of third molar techniques for age estimation applied to Brazilian adolescents—a systematic review. Research, Society and Development 2020; 9:e9339109395.

[42] Mahmud S, Hossain MF, Muyeed A, et al Risk assessment and clinical implications of COVID-19 in multiple myeloma patients: a systematic review and meta-analysis. PLoS One 2024; 19:e0308463. 39241024 10.1371/journal.pone.0308463 PMC11379232

[43] Nanayakkara D, Liyanapathirana V, Kandauda C, Gihan C, Ekanayake A, Adasooriya D. Maternal vaginal colonization with selected potential pathogens of neonatal sepsis in the era of antimicrobial resistance, a single center experience from Sri Lanka. BMC Infect Dis 2018; 18:351. 30055584 10.1186/s12879-018-3262-y PMC6064104

[44] Rakotondrasoa A, Passet V, Herindrainy P, et al Characterization of *Klebsiella pneumoniae* isolates from a mother-child cohort in Madagascar. J Antimicrob Chemother 2020; 75:1736–46. 32303060 10.1093/jac/dkaa107

[45] Meredith HR, Kularatna S, Nagaro K, et al Colonization with multidrug-resistant Enterobacteriaceae among infants: an observational study in Southern Sri Lanka. Antimicrob Resist Infect Control 2021; 10:72. 33931120 10.1186/s13756-021-00938-3 PMC8086278

[46] Villinger D, Schultze TG, Musyoki VM, et al Genomic transmission analysis of multidrug-resistant gram-negative bacteria within a newborn unit of a Kenyan tertiary hospital: a four-month prospective colonization study. Front Cell Infect Microbiol 2022; 12:892126. 36093198 10.3389/fcimb.2022.892126 PMC9452910

[47] Bah SY, Kujabi MA, Darboe S, et al Acquisition and carriage of genetically diverse multi-drug resistant gram-negative bacilli in hospitalised newborns in The Gambia. Commun Med (Lond) 2023; 3:79. 37270610 10.1038/s43856-023-00309-6 PMC10239441

[48] Dutta S, Chakraborty A, Biswal M, Sharma A, Suri V, Ray P. Multidrug-resistant, potentially pathogenic bacteria in mother's milk, and neonatal oral and rectal swabs of preterm mother-neonate dyads. Indian J Microbiol 2024; 65:2004–14 41424889 10.1007/s12088-024-01413-4 PMC12712266

[49] Dos Santos S, Moussounda M, Togola M, et al Carbapenem-producing Enterobacteriaceae in mothers and newborns in southeast Gabon, 2022. Front Cell Infect Microbiol 2024; 14:1341161. 38390622 10.3389/fcimb.2024.1341161 PMC10881798

[50] Njeuna A, Founou LL, Founou RC, et al High prevalence and genetic diversity of multidrug-resistant and extended-spectrum ß-lactamase–producing *Escherichia coli* and *Klebsiella pneumoniae* in mothers and neonates in a Cameroonian labor ward. Am J Infect Control 2024; 52:1273–82. 38876168 10.1016/j.ajic.2024.06.002

[51] Okomo UA, Darboe S, Bah SY, et al Maternal colonization and early-onset neonatal bacterial sepsis in the Gambia, West Africa: a genomic analysis of vertical transmission. Clin Microbiol Infect 2023; 29:386.e1–e9. 10.1016/j.cmi.2022.10.012 36243352

[52] Cutland CL, Madhi SA, Zell ER, et al Chlorhexidine maternal-vaginal and neonate body wipes in sepsis and vertical transmission of pathogenic bacteria in South Africa: a randomised, controlled trial. Lancet 2009; 374:1909–16. 19846212 10.1016/S0140-6736(09)61339-8

[53] World Health Organization . Kangaroo mother care: a practical guide. **2003**. Available at: https://www.who.int/publications/i/item/9241590351.

[54] Delerue T, de Pontual L, Carbonnelle E, Zahar JR. The potential role of microbiota for controlling the spread of extended-spectrum beta-lactamase–producing Enterobacteriaceae (ESBL-PE) in neonatal population. F1000Res 2017; 6:1217. 28781766 10.12688/f1000research.10713.1 PMC5531162

[55] Nadimpalli ML, Bourke CD, Robertson RC, Delarocque-Astagneau E, Manges AR, Pickering AJ. Can breastfeeding protect against antimicrobial resistance? BMC Med 2020; 18:392. 33317529 10.1186/s12916-020-01862-w PMC7737306

[56] Parm Ü, Tiit-Vesingi A, Soeorg H, et al Effect of early directed implementation of family-integrated care measures on colonisation with Enterobacteriaceae in preterm neonates in NICU. BMJ Paediatr Open 2023; 7:e001712. 10.1136/bmjpo-2022-001712 PMC10193046 37192777

