# Supplementary material for: Molecular Relatedness of Maternal and Neonatal Multidrug-Resistant Gram-Negative Colonization Isolates in Low- and Middle-Income Countries: A Systematic Review
Source: Open Forum Infect Dis. 2026 Jan 14;13(2):ofag010. doi: 10.1093/ofid/ofag010 (PMC12871433; doi:10.1093/ofid/ofag010)
Supplement: ofag010_Supplementary_Data [file ofag010_supplementary_data.zip › Appendix 2 JBI Assessment (1).docx]

Appendix 2: Joanna Briggs Institute (JBI) Assessment of Article Quality and Potential for Bias.

| **Cross-Sectional Design** | 1 | 2 | 3 | 4 | 5 | 6 | 7 | 8 | Included Applicable Items | Overall Assessment of Quality |
| --- | --- | --- | --- | --- | --- | --- | --- | --- | --- | --- |
| Nanayakkara 2018 | Y | Y | Y | Y | N | N | Y | Y | 75% | Good |
| Carvahlo 2022 | Y | Y | Y | Y | Y | Y | Y | Y | 100% | Good |
| Chomkatekaew 2023 | Y | Y | Y | Y | N | N | Y | Y | 75% | Good |
| Mayanja 2023 | Y | Y | Y | Y | N | N | Y | N | 62% | Moderate |
| Dos Santos 2024 | Y | Y | Y | Y | N/A | N/A | Y | Y | 100% | Good |
| Njeuna 2024 | Y | Y | Y | Y | Y | N | Y | Y | 87.00% | Good |

1. Were the criteria for inclusion in the sample clearly defined?
2. Were the study subjects and the setting described in detail?
3. Was the exposure measured in a valid and reliable way?
4. Were objective, standard criteria used for measurement of the condition?
5. Were confounding factors identified?
6. Were strategies to deal with confounding factors stated?
7. Were the outcomes measured in a valid and reliable way?
8. Was appropriate statistical analysis used?

| **Cohort Design** | 1 | 2 | 3 | 4 | 5 | 6 | 7 | 8 | 9 | 10 | 11 | Included Applicable Items | Overall Assessment of Quality |
| --- | --- | --- | --- | --- | --- | --- | --- | --- | --- | --- | --- | --- | --- |
| Rakotondrasoa 2020 | Y | Y | Y | N | N | N/A | Y | Y | Y | N/A | Y | 77% | Good |
| Meredith 2021 | Y | Y | Y | Y | Y | N/A | Y | Y | N | Y | Y | 90% | Good |
| Villinger 2022 | Y | Y | Y | N | N | N/A | Y | Y | Y | N/A | Y | 77% | Good |
| Bah 2023 | Y | Y | Y | N | N | N/A | Y | Y | Y | N/A | N | 66% | Moderate |
| Dutta 2024 | Y | Y | Y | Y | N | N/A | Y | N | N | N | Y | 60% | Moderate |

1. Were the two groups similar and recruited from the same population?
2. Were the exposures measured similarly to assign people to both exposed and unexposed groups?
3. Was the exposure measured in a valid and reliable way?
4. Were confounding factors identified?
5. Were strategies to deal with confounding factors stated?
6. Were the groups/participants free of the outcome at the start of the study (or at the moment of exposure)?
7. Were the outcomes measured in a valid and reliable way?
8. Was the follow up time reported and sufficient to be long enough for outcomes to occur?
9. Was follow up complete, and if not, were the reasons to loss to follow up described and explored?
10. Were strategies to address incomplete follow up utilized?
11. Was appropriate statistical analysis used?
